# Use, physiological effects and safety of pulmonary vasodilator therapy in thoracic surgical patients: a scoping review protocol

**DOI:** 10.1136/bmjopen-2026-123437

**Published:** 2026-10-08

**Authors:** Gareth Lipton, Philip McCall, Alastair Burden, Benjamin Shelley

**Affiliations:** 1 Clinical Research Fellow in Anaesthesia, University of Glasgow School of Medicine Dentistry and Nursing, Glasgow, UK; 2 Cardiothoracic Anaesthesia & Critical Care, Golden Jubilee National Hospital West of Scotland Regional Heart and Lung Centre, Glasgow, UK; 3 University of Glasgow School of Medicine Dentistry and Nursing, Glasgow, UK; 4 Anaesthesia, Queen Elizabeth University Hospital, Glasgow, UK; 5 New Lister Building, University of Glasgow School of Medicine Dentistry and Nursing, Glasgow, UK

**Keywords:** Thoracic surgery, Hemodynamics, ANAESTHETICS, Respiratory physiology, CLINICAL PHARMACOLOGY

## Abstract

**Abstract:**

**Introduction:**

Thoracic surgery requiring one-lung ventilation is associated with acute increases in right ventricular (RV) afterload, which may contribute to perioperative RV dysfunction and long-term functional limitation. Pulmonary vasodilator therapy represents a potential strategy to attenuate these effects however, the breadth, characteristics and implications of pulmonary vasodilator therapy in the thoracic surgical population have not been comprehensively synthesised. This scoping review aims to map the existing evidence base on the use, physiological effects and safety of pulmonary vasodilator therapy in adult thoracic surgery.

**Methods and analysis:**

This protocol has been developed in accordance with Joanna Briggs Institute (JBI) methodology and reported in line with the PRISMA Extension for Scoping Reviews (PRISMA-ScR). A comprehensive search of Ovid MEDLINE and Embase will be conducted from 1946 to 2026. Eligible studies will include original research (including case reports and case series) involving adult patients (≥18 years) undergoing thoracic surgery with perioperative administration of pulmonary vasodilator or inodilator therapy. Cardiac surgery, transplantation, non-surgical populations and paediatric studies will be excluded. Two independent reviewers will perform study selection, with disagreements resolved by consensus or third reviewer adjudication. Data extraction will be conducted by one reviewer and independently verified by a second. Extracted data will include study characteristics, patient population, surgical context, pharmacological interventions and reported physiological and safety outcomes. Data will be summarised descriptively using tabulated and narrative synthesis. Consistent with scoping review objectives, formal critical appraisal will not be undertaken, as the aim is to map the evidence rather than assess effect size or certainty. Methodological characteristics will be described to contextualise interpretation of results.

**Ethics and dissemination:**

Ethical approval is not required. Findings will be disseminated through peer-reviewed publication and conference presentation, informing future clinical trial design.

**STRENGTHS AND LIMITATIONS OF THIS STUDY**

- This protocol follows established Joanna Briggs Institute methodology and Preferred Reporting Items for Systematic reviews and Meta-Analyses extension for Scoping Reviews reporting standards, enhancing transparency and reproducibility.
- A comprehensive, librarian-supported search strategy across MEDLINE and Embase improves the likelihood of capturing relevant literature.
- Inclusion of a broad range of study designs, including case reports and series, allows comprehensive mapping of an emerging and heterogenous evidence base.
- The absence of a formal risk-of-bias assessment may limit interpretation of the quality and certainty of the evidence mapped.
- Restriction to English-language and published studies (excluding grey literature) may introduce language and publication bias.

## Rationale

 Lung cancer is a leading cause of cancer death in the UK^1–3^ and worldwide.^4^ Lung resection represents the best chance of cure,^5^ with minimally-invasive (thoracoscopic) techniques typically preferred due to improved peri- and postoperative patient outcomes.^6^ Thoracoscopic lung resection involves general anaesthesia, one-lung ventilation (OLV), capnothorax, lateral positioning, surgical pulmonary parenchymal manipulation and pulmonary vascular clamping. Physiological responses include hypoxic pulmonary vasoconstriction, increased intrathoracic pressures and acute redirection of blood flow from the operative to non-operative lung. Marked pulmonary vascular and right ventricular (RV) haemodynamic effects are observed, including acutely elevated pulmonary vascular resistance, RV and pulmonary arterial pressures, and hence RV afterload.^7^ RV dysfunction, however, occurs frequently, and is identifiable using both transthoracic echocardiography^8^ and cardiac MRI.^9^ This dysfunction may result from an acute sterile inflammatory cardiomyopathy^10^ occurring in response to acutely increased afterload.^11^ The observed acute elevation in RV afterload is analogous to other pathological states including acute pulmonary embolism (PE).^12^ Animal models of PE demonstrate upregulation of inflammatory genes, cytokines, chemokines and cells,^13 14^ in association with persistently impaired RV function, even after afterload has returned to baseline,^15^ and in the absence of RV myocardial ischaemia.

Research supports the hypothesis that the adaptive/maladaptive sequelae of this acutely elevated RV afterload results in long-term RV dysfunction, manifest as exercise limitation and dyspnoea.^16–19^ There are currently no licensed targeted pharmacotherapies which attenuate acute RV afterload elevation in this context. All patients undergoing lung resection are thus subjected to a homogenous, controlled, iatrogenic RV insult. A central hypothesis to our programme of research is that by attenuating acute increases in RV afterload perioperatively, pulmonary vasodilating agents may prevent RV injury and ameliorate disabling long-term functional limitation.

Therapeutic pulmonary vasodilation is a mainstay of cardiopulmonary protection (and improved oxygenation) in select patient groups at risk of heightened pulmonary vascular resistance both in acute and chronic contexts, including pulmonary hypertension, heart and lung transplantation, acute respiratory distress syndromes and patients with congenital cardiac disease.^20^ Pulmonary vasodilators are available in a range of drug classes and routes of administration, with variable extra-pulmonary effects.

To date, there has been no comprehensive synthesis of evidence specifically addressing perioperative RV afterload modulation in thoracic surgery. Therefore, we propose to perform a scoping review to map the evidence base of this potential therapeutic target. Forthwith, *thoracic surgery* excludes cardiac and mediastinal surgery.

The intended surgical population of this review is those associated with acutely elevated RV afterload, namely lung resection and other surgeries entering the thoracic cavity that involve one-lung ventilation. Patients undergoing one-lung ventilation for non-pulmonary intrathoracic surgery (eg, oesophagectomy) are subjected to a similar physiological insult,^21^ so are considered in this review to broaden the scope of available evidence. Pulmonary vasodilator therapy in thoracic organ transplantation,^22 23^ cardiac surgery^24–26^ and congenital cardiac surgery^27^ is well described, with markedly different physiological insults and invariable exposure to cardiopulmonary bypass, so are not considered in this review. Similarly, patients not undergoing surgery and children are not considered due to heterogeneity of pathophysiology and aetiology (primarily chronic disease or indicated to improve oxygenation rather than attenuate RV afterload increases).^20^ Inhaled pulmonary dilator therapy in acute pulmonary embolism has been reviewed previously.^28^

Scoping review methodology will permit mapping of concepts, types of evidence and current knowledge gaps prior to designing clinical trials in this field. Scoping review methodology may reveal key safety outcomes/effects—required for clinical trial development—including: dose-related, non-dose-related, delayed, failure of therapy and clinical metrics. Outputs will inform dosing interventions, dosing strategies and endpoint selection. In parallel to this scoping review, our group is conducting a single centre, prospective, observational clinical feasibility study to measure continuous perioperative pulmonary/RV haemodynamics in patients undergoing lung resection as a pathway to a clinical trial of pulmonary vasodilator therapy in lung resection (NCT07812207 - Not yet recruiting).

## Objectives

To map and characterise the use, physiological effects and safety of pulmonary vasodilator therapy in adult thoracic surgery.

We will systematically follow the Joanna Briggs Institute (JBI) updated guidelines for scoping reviews to:

- Map and categorise the available literature on pulmonary vasodilator therapy in adult thoracic surgery.
- Characterise the pharmacological agents, routes of administration and clinical contexts in which these therapies are used.
- Describe reported physiological outcomes, endpoints and methodological approaches across studies.
- Identify patient subgroups and clinical contexts associated with potential benefit or harm and highlight gaps to inform future research.

### Review questions

#### Primary question

1. What roles, applications and reported physiological and safety outcomes of pulmonary vasodilator therapy are described in adult thoracic surgery?

#### Secondary questions

1. What types of studies are available?
2. Which vasodilator drugs are used?
  - Which pharmacological classes and specific agents are used?
  - What routes and dosing strategies are used?
3. In what therapeutic contexts are pulmonary vasodilators used, and how are they administered?
4. What pharmacodynamic or clinical endpoints are measured?
5. What evidence exists regarding impact on right ventricular afterload?
6. Where are the knowledge gaps in this field?

## Methods and analysis

### Protocol and registration

This scoping review protocol has been developed in line with JBI guidelines,^29 30^ and assessed for transparency, completeness and accuracy in reporting using the updated ‘PRISMA extension for scoping reviews: (PRISMA-ScR): checklist and explanation’.^31^ The *RightReview* tool confirmed appropriateness of Scoping Review methodology for this subject field.^32^ Prior to registration, The International Prospective Register of Systematic Reviews (PROSPERO) and PubMed were searched, confirming that no review articles on this subject are registered or published. Scoping reviews are ineligible for PROSPERO registration. This protocol was registered on Open Science Framework (OSF) (OSF Registration DOI: 10.17605/OSF.IO/WAB5N) on 29 May 2026 (OSF Associated Project https://osf.io/pzr57).

### Ethics

Ethical approval is not required to conduct this scoping review which will present only previously published data.

### Patient and public involvement

We have formed a patient and public involvement group of patient partners who have undergone lung resection surgery. We will engage partners in an iterative manner, offering input into the interpretation of outcomes and the dissemination of findings. This group is involved in our wider programme of research into the impact of thoracic surgery on the right ventricle.

### Eligibility criteria

This scoping review will evaluate all available literature on pulmonary vasodilators/inodilators in adult patients undergoing thoracic surgery, with criteria for inclusion and exclusion presented in table 1.

**Table 1:** Scoping review inclusion and exclusion criteria

	Inclusion criteria	Exclusion criteria
Population	Adults (≥18 years) Human only Elective (planned) or emergency (unplanned) surgery Lung resection and/or surgeries entering the thoracic cavity involving one-lung ventilation Thoracotomy or thoracoscopic surgery Upper gastrointestinal surgeries will be included where the thoracic cavity is entered	Lung transplant surgery Cardiac surgery Surgeries involving cardiopulmonary bypass No surgery performed Thoracostomy tube insertion as sole thoracic procedure
Concept	Perioperative administration of any pulmonary vasodilators/inodilators via any route Perioperative defined as administration within 48 hours of surgery/procedure Uptitrated dose or introduction of new pulmonary vasodilator in the perioperative period in patients on established chronic pulmonary hypertension treatment	Studies involving administration of pulmonary vasodilators/inodilators in patients not undergoing surgery Inhaled oxygen as the sole pulmonary vasodilator agent Unchanged perioperative continuation of pulmonary vasodilators for chronic pulmonary hypertension Studies involving secondary pulmonary vasodilating agents where no right ventricular or pulmonary vascular haemodynamic measures are reported
Context	Cardiopulmonary physiological change, adverse systemic or physiological therapeutic effects	
Sources	Original research including case reports and case series 1946–2026	Review articles Abstracts not available in English
Inclusion and exclusion criteria formatted according to the JBI-recommended PCC (Population, Concept, Context) framework.		

#### Population

Adult (≥18 years old) humans undergoing planned (elective) and unplanned (emergency) thoracic surgery using either thoracotomy or thoracoscopy. For the purposes of the review, thoracic surgery will be defined as: lung resection and other surgeries entering the thoracic cavity that involve one-lung ventilation (also termed *lung isolation* and *selective pulmonary ventilation*). Studies involving mixed populations will be eligible if extractable data meets the inclusion criteria.

#### Concept

Thoracic surgery and one-lung ventilation cause acutely elevated right ventricular afterload. Upper gastrointestinal surgery—where the thoracic cavity is entered—exerts a similar physiological effect. Pulmonary vasodilators (including inodilators) may attenuate this effect, in a cardioprotective manner. Physiological pharmacodynamic endpoints are incompletely defined. Pulmonary vasodilator therapy may be selective (acting only on pulmonary vasculature) or non-selective (resulting in pulmonary and systemic effects). Indications include targeting improvements in pulmonary and right ventricular haemodynamics and oxygenation. Adverse effects are expected to occur more commonly with systemic administration and with agents with longer half-lives.

Pulmonary vasodilators are defined as drugs which primarily act on the pulmonary vasculature with a vasodilatory effect and include pulmonary inodilators. Given the relative novelty of this therapeutic approach (modulating pulmonary vascular physiology) in the context of thoracic surgery, drugs which have a secondary pulmonary vasodilatory effect (eg, Dobutamine)—which may be considered to have an alternative primary effect (eg, positive inotropy)—are included provided haemodynamic outcomes measures of the right ventricular-pulmonary vascular unit are reported. Where records report the use of secondary pulmonary vasodilating drugs in adults undergoing one-lung ventilation but no right ventricular or pulmonary vascular haemodynamic measures are reported (eg, modifications to oxygenation only), they will be excluded during screening. All routes of administration will be included, including (but not limited to): inhaled, oral or intravenous (peripheral or central) route. Classes/drugs included (but not limited to) are:

- Phosphodiesterase-3 and -5 inhibitors (eg, Milrinone, Enoximone, Sildenafil, Tadalafil).
- Inhaled nitric oxide.
- Prostacyclin analogues (eg, Epoprostenol, Iloprost, Treprostinil).
- Endothelin receptor antagonists (eg, Bosentan).
- Calcium sensitisers (eg, Levosimendan).
- Beta-1 adrenoceptor agonists (eg, Dobutamine, Dopexamine, Epinephrine, Dopamine).

#### Context

The physiological insult described occurs during one-lung ventilation (OLV: indicated for lung isolation, surgical access and differential ventilation) using a double-lumen endotracheal tube, bronchial blocker or single lumen tube inserted endobronchially with mechanical ventilation and lung resection (which may occasionally be performed in spontaneously ventilating patients). There already exists substantial literature on therapeutic/rescue use of pulmonary vasodilators in cardiac surgery with cardiopulmonary bypass, neonatal respiratory distress, acute respiratory distress syndrome and pulmonary hypertension. This scoping review will not include these conditions, as they are significantly different pathophysiological entities. Beta-1 adrenoceptor agonists are systemic positive inotropes/vasoactive agents, with secondary pulmonary vasodilating effects. These properties may influence outcomes in the context of attenuating OLV-induced RV afterload elevation. Beta-1 agonists are therefore included for comprehensiveness.

#### Exclusion criteria

Animal studies will be excluded due to limited applicability of pharmacotherapy to human physiology. Cardiac surgery and cardiopulmonary bypass cases will be excluded due to significant differences in drug indication and pathophysiology. Non-adults will be excluded for the following reasons: lung resection and OLV are rare in under 18-year-olds, indications for OLV are different in children and adults, those children undergoing lung resection and/or OLV tend to have other significant co-morbidity which may be associated with cardiopulmonary physiological differences. Patients receiving pulmonary vasodilator therapy outwith surgery (eg, medical management of chronic pulmonary hypertension) will be excluded. Thoracostomy tube insertion for pleural drainage as sole thoracic procedure is excluded as this falls outwith the context of major pulmonary resection. Thoracostomy is included as a search term due to variable description in the literature and in select cases where associated with additional surgical entry to the thoracic cavity and/or OLV, to enable identification of relevant physiological studies.

### Information sources

OVID Medline & Embase (1946–2026) will be searched for human clinical studies (observational and interventional) of pulmonary vasodilator/inodilator therapy in adult thoracic surgery as outlined within the search strategy. These databases were selected as they provide broad coverage of biomedical and pharmacological literature relevant to anaesthesia. A pilot search of Cochrane CENTRAL retrieved no relevant results that met with the objectives of this scoping review therefore this database was not included. The most recent pilot search prior to protocol registration was performed in May 2026.

### Search

A comprehensive search strategy has been developed, which accounts for the heterogenous terminology and lack of strict taxonomy within this field. A pilot search of Medline & Embase (via OVID) was performed to identify relevant key words and terms for development of the full search strategy. The reference list of obtained results will be examined for additional references. Efforts will be made to contact by email corresponding authors of unobtainable but potentially eligible papers identified during the search process. Full final search strategies, developed with support from information scientists (ER, AW and KC), are included as appendices to this protocol (online supplemental appendices 2–3).

### Selection of sources of evidence

Referenced results will be merged using EndNote 21 (Clarivate) software. Covidence (Veritas Health Innovation, Melbourne, Australia) software will be used to de-duplicate and screen results. Two independent authors (GL and AB) will screen for inclusion by title and abstract with disagreement arbitration by one of two independent subject-specialist co-authors (BS and PM). All abstracts selected for inclusion will be full text screened by inclusion/exclusion criteria by two independent authors. Authors will meet to consider disagreements or uncertainties in this secondary screening process. In the event of disagreement or uncertainty in this secondary screening process, a third subject-specialist reviewer will adjudicate. The systematic literature search will be represented using a Preferred Reporting Items for Systematic reviews and Meta-Analyses extension for Scoping Reviews (PRISMA-ScR) flowchart.

### Data charting process and data items

Basic descriptive (eg, lead author(s), publication year, country, study participants, surgery, pulmonary vasodilator(s) used, pharmacological class, indication for treatment (eg, hypoxaemia, elevated pulmonary artery pressure), intended therapeutic use (eg, prophylactic, therapeutic, rescue), route of administration, outcome measure and study type) data will be tabulated. This will be piloted following initial identification of key papers to test feasibility for final review and refined as necessary prior to final data extraction on identification of literature for review. The pilot data extraction tool (online supplemental appendix 1: Table 1) may be amended following planned piloting on a purposive sample—20% of included studies—in line with JBI guidance, an iterative process to enhance capture of variables required before commencing full extraction. The final completed tool—and amendments, if present—will be included in the scoping review manuscript. Data extraction will be conducted by the lead author (GL), and independently verified by co-authors (AB, BS, PM). Disagreements in extraction processes will be discussed as a group until consensus is reached.

### Critical appraisal

Critical appraisal will not be performed, consistent with scoping review methodology.

### Synthesis of results

An overview and description of results will be reported in line with JBI guidance, providing statistical, tabulated representation (evidence mapping stratified by drug class, route of administration and surgical context) and narrative description. Scoping review methodology requires presentation of conceptual categories, which may be best presented using mixed methods. As an iterative process with expected heterogeneous papers under review, final data presentation format is likely to evolve as results are obtained.

### Anticipated protocol deviations

This scoping review will be conducted according to established methodological guidelines. As an iterative process, protocol deviation may be necessary during the search strategy, data extraction or data presentation stages. To maintain transparency, protocol deviations will be clearly detailed and justified in the final scoping review manuscript.

## Summary

This protocol details how a scoping review to investigate the use, effects and safety of pulmonary vasodilator therapy in thoracic surgery to attenuate perioperative right ventricular afterload elevation will be conducted. The outcomes will inform a subsequent clinical trial of pulmonary vasodilators in thoracic surgery.

### Strengths and limitations

This review will be conducted systematically, informed by the Preferred Reporting Items for Systematic reviews and Meta-Analyses extension for Scoping Reviews (PRISMA-ScR) guidelines. We expect to identify literature across a heterogeneous range of surgical procedures, with different pulmonary vasodilator classes, routes, dosage, timing and indications. Outcome measures are poorly defined. This may limit direct comparability but provide an opportunity to map the current evidence and identify knowledge gaps. Most published literature involving pulmonary vasodilator therapy falls within the realms of our exclusion criteria (non-surgical or cardiac surgical), so we expect a high exclusion rate. Abstracts and full texts not available in English will be excluded due to resource constraints and lack of translation capacity, accepting that this has the potential to introduce language bias. Grey literature (eg, conference abstracts, protocols, academic theses, expert opinion, policy documents) will be excluded for feasibility reasons, high duplication risk and anticipated lack of extractable physiological data, which would impair mapping of pharmacodynamic outcomes.

Finally, the search strategy aims to comprehensively incorporate all clinically relevant pulmonary vasodilators and thoracic surgical procedures within the remit of this scoping review.

### Dissemination plan

Following completion, we expect to publish results in a peer-reviewed journal and present nationally. Findings will inform dose selection, endpoint definition and feasibility parameters for future interventional trials.

## Supplementary material

10.1136/bmjopen-2026-123437 online supplemental file 1

10.1136/bmjopen-2026-123437 online supplemental file 2

10.1136/bmjopen-2026-123437 online supplemental file 3

## References

[1] Office for National Statistics (2024). Death registration summary statistics, England and Wales: 2025.

[2] NICR (2025). All cancers (ex NMSC): 1993-2022.

[3] Cancer Research UK (2025). Lung cancer statistics: lung cancer incidence. https://www.cancerresearchuk.org/health-professional/cancer-statistics/statistics-by-cancer-type/lung-cancer#lung_stats0.

[4] Leiter A, Veluswamy RR, Wisnivesky JP (2023). The global burden of lung cancer: current status and future trends. Nat Rev Clin Oncol.

[5] Fu F, Chen Z, Chen H (2023). Treating lung cancer: defining surgical curative time window. Cell Res.

[6] Cheng X, Onaitis MW, D’amico TA (2018). Minimally Invasive Thoracic Surgery 3.0: Lessons Learned From the History of Lung Cancer Surgery. Ann Surg.

[8] Shelley B, Glass A, Keast T (2023). Perioperative cardiovascular pathophysiology in patients undergoing lung resection surgery: a narrative review. Br J Anaesth.

[8] McErlane J, Glass A, Soosay A (2023). Feasibility of right ventricular free wall longitudinal strain assessment of contractile reserve in patients undergoing lung resection. Br J Anaesth.

[9] McCall PJ, Arthur A, Glass A (2019). The right ventricular response to lung resection. J Thorac Cardiovasc Surg.

[10] Murphy E, Glass A, McCall P (2021). Myocardial inflammation after major non-cardiac thoracic surgery. Br J Anaesth.

[11] Glass A, McCall P, Arthur A (2023). Pulmonary artery wave reflection and right ventricular function after lung resection. Br J Anaesth.

[12] Elrakhawy HM, Alassal MA, Shaalan AM (2018). Impact of Major Pulmonary Resections on Right Ventricular Function: Early Postoperative Changes. Heart Surg Forum.

[13] Watts JA, Gellar MA, Stuart LK (2009). Proinflammatory events in right ventricular damage during pulmonary embolism: effects of treatment with ketorolac in rats. J Cardiovasc Pharmacol.

[14] Watts JA, Marchick MR, Kline JA (2010). Right ventricular heart failure from pulmonary embolism: key distinctions from chronic pulmonary hypertension. J Card Fail.

[15] Greyson C, Xu Y, Cohen J (1997). Right ventricular dysfunction persists following brief right ventricular pressure overload. Cardiovasc Res.

[16] Matyal R, Mahmood F, Hess P (2010). Right ventricular echocardiographic predictors of postoperative supraventricular arrhythmias after thoracic surgery: a pilot study. Ann Thorac Surg.

[17] Kowalewski J, Brocki M, Dryjański T (1999). Right ventricular morphology and function after pulmonary resection. Eur J Cardiothorac Surg.

[18] Pedoto A, Amar D (2009). Right heart function in thoracic surgery: role of echocardiography. Curr Opin Anaesthesiol.

[19] Lewis JW, Bastanfar M, Gabriel F (1994). Right heart function and prediction of respiratory morbidity in patients undergoing pneumonectomy with moderately severe cardiopulmonary dysfunction. J Thorac Cardiovasc Surg.

[20] Liu K, Wang H, Yu S-J (2021). Inhaled pulmonary vasodilators: a narrative review. Ann Transl Med.

[21] Trepte C, Haas S, Meyer N (2012). Effects of one-lung ventilation on thermodilution-derived assessment of cardiac output. Br J Anaesth.

[22] Ghadimi K, Cappiello J, Cooter-Wright M (2022). Inhaled Pulmonary Vasodilator Therapy in Adult Lung Transplant: A Randomized Clinical Trial. JAMA Surg.

[23] Krebs R, Morita Y (2020). Inhaled Pulmonary Vasodilators and Thoracic Organ Transplantation: Does Evidence Support Its Use and Cost Benefit?. *Semin Cardiothorac Vasc Anesth*.

[24] David N, Lakha S, Walsh S (2024). Novel inhaled pulmonary vasodilators in adult cardiac surgery: a scoping review. Can J Anaesth.

[25] Foote HP, Hornik CP, Hill KD (2022). A systematic review of clinical study evidence for pulmonary vasodilator therapy following surgery with cardiopulmonary bypass in children with CHD. Cardiol Young.

[26] Sardo S, Tripodi VF, Guerzoni F (2023). Pulmonary Vasodilator and Inodilator Drugs in Cardiac Surgery: A Systematic Review With Bayesian Network Meta-Analysis. J Cardiothorac Vasc Anesth.

[27] Ollosu M, Tripodi VF, Aresu C (2026). Efficacy and Safety of Pulmonary Vasodilator and Inodilator Drugs in Congenital Heart Disease Surgery: A Systematic Review and Network Meta-Analysis of Randomized Controlled Trials. J Cardiothorac Vasc Anesth.

[28] Lyhne MD, Kline JA, Nielsen-Kudsk JE (2020). Pulmonary vasodilation in acute pulmonary embolism - a systematic review. Pulm Circ.

[29] Peters MDJ, Godfrey C, McInerney P (2022). Best practice guidance and reporting items for the development of scoping review protocols. JBI Evid Synth.

[30] Pollock D, Peters MDJ, Khalil H (2023). Recommendations for the extraction, analysis, and presentation of results in scoping reviews. JBI Evid Synth.

[31] Tricco AC, Lillie E, Zarin W (2018). PRISMA Extension for Scoping Reviews (PRISMA-ScR): Checklist and Explanation. Ann Intern Med.

[32] Amog K, Pham B, Courvoisier M (2022). The web-based “Right Review” tool asks reviewers simple questions to suggest methods from 41 knowledge synthesis methods. J Clin Epidemiol.

