# Supplementary material for: Use, physiological effects and safety of pulmonary vasodilator therapy in thoracic surgical patients: a scoping review protocol
Source: BMJ Open. 2026 Oct 8;16(10):e123437. doi: 10.1136/bmjopen-2026-123437 (PMC13656151; doi:10.1136/bmjopen-2026-123437)
Supplement: Supplementary file 1 [file bmjopen-2026-123437.reviewer_comments.pdf]

PEER REVIEW HISTORY

BMJ Open publishes all reviews undertaken for accepted manuscripts. Reviewers are asked to complete a checklist review form and are provided with free text boxes to elaborate on their assessment. These free text comments are reproduced below.

ARTICLE DETAILS

Title (Provisional)

Use, physiological effects and safety of pulmonary vasodilator therapy in thoracic surgical patients: a scoping review protocol

Authors

Lipton, Gareth; McCall, Philip; Burden, Alastair; Shelley, Benjamin

---

VERSION 1 - REVIEW

**Reviewer**	**1**
**Name**	**Lampridis, Savvas**
**Affiliation**	**Imperial College London National Heart and Lung Institute, Faculty of Medicine**
**Date**	**22-Jul-2026**
**COI**	**None**

I was pleased to receive your manuscript as a reviewer. I found the topic clinically relevant and the translational rationale interesting. A few comments/queries:

1. I think the scope needs to be clearer. Your rationale centres on lung resection and one-lung ventilation, but the eligibility criteria also include upper GI surgery and even procedures without one-lung ventilation. What is the intended surgical population?

2. I'd reconsider including thoracostomy, which refers to pleural drainage. Did you mean thoracotomy?

3. The intervention definition seems too broad to me. You include systemic vasoactive drugs alongside targeted pulmonary vasodilators. I suggest including these agents only when they were used to modify pulmonary haemodynamics or RV function.

4. Please define "perioperative administration". What time window will you accept, and will chronic pulmonary hypertension treatment simply continued during admission be eligible? I'd also distinguish prophylactic, therapeutic, and rescue use.

5. I'm a bit concerned that the outcome block in the search strategy may exclude relevant studies. Have you validated the strategy against known eligible studies? I'd consider removing this block.

6. Riociguat is listed as eligible but does not appear in the search. I guess "betaprost" is a typo for beraprost. The appendices should present the final searches rather than pilot versions.

7. Please state how you'll handle non-English full texts, conference abstracts, protocols, and theses.

8. Please confirm that two reviewers will assess all full texts independently. I'd avoid requiring unanimous agreement, as this may unintentionally favour exclusion. Instead, use third-reviewer adjudication.

9. I think the data-charting form is too limited for your objectives. I'd add baseline pulmonary hypertension and RV status, operation and resection extent, one-lung ventilation details, treatment indication, dose, timing, duration, comparator, outcome timing, adverse events.

Thank you.

VERSION 1 - AUTHOR RESPONSE

Reviewer's comments to author

General response: Thank you for taking the time to review this manuscript and offering your expert feedback. We are grateful for the rigour with which you have appraised our submission. We are also pleased to note that the reviewer finds the topic clinically relevant and the translational rationale interesting.

In response to the reviewer's comments/queries:

1. **Comment:** *I think the scope needs to be clearer. Your rationale centres on lung resection and one-lung ventilation, but the eligibility criteria also include upper GI surgery and even procedures without one-lung ventilation. What is the intended surgical population?*

Response: Thank you for highlighting this important point.

The primary insults we hypothesise to cause acutely elevated RV afterload with resultant postoperative RV dysfunction are one-lung ventilation and

thoracic/thoracoscopic surgery (multifactorial: surgical pulmonary parenchymal manipulation and/or resection, capnothorax, pulmonary arterial clamping,). Therefore, we aimed to include all non-cardiac surgical procedures using one-lung ventilation and/or entering the thoracic cavity. Upper GI surgery (e.g. oesophagectomy) often involves OLV and therefore subjects patients to a similar physiological insult.

We have increased the clarity within our Rationale and Concept sections to address this.

The manuscript now reads (forthwith; amended/new text in red, unchanged text in **black**):

- Page 7, lines 118-120:
    - The intended surgical population of this review is those associated with acutely elevated RV afterload, namely one-lung ventilation and and/or those entering the thoracic cavity. Patients undergoing one-lung ventilation for non-pulmonary intrathoracic surgery (e.g. oesophagectomy) are subjected to a similar physiological insult…
- Page 13, lines 203-205:
    - Thoracic surgery and one-lung ventilation cause acutely elevated right ventricular afterload. Upper gastrointestinal surgery - where the thoracic cavity is entered - exerts a similar physiological effect. Pulmonary vasodilators…

2. **Comment:** *I'd reconsider including thoracostomy, which refers to pleural drainage. Did you mean thoracotomy?*

Response: Thank you for raising this point. The inclusion criteria have been amended accordingly, such that if the sole "surgical procedure" is thoracostomy tube insertion (provided the record meets the other criteria) then this will be excluded during title/abstract/full text review. Figure 1 within the amended manuscript has been changed such that "thoracostomy" has been removed from the inclusion criteria and "thoracostomy tube insertion as sole thoracic procedure" has been added to the exclusion criteria. "Thoracostomy" is only included as a search term due to variable description in the literature and in select cases where associated with additional surgical entry to the thoracic cavity and/or OLV, to enable identification of relevant physiological studies.

The manuscript now reads:

- Page 15, lines 247-253:
  - Patients receiving pulmonary vasodilator therapy outwith surgery (e.g. medical management of chronic pulmonary hypertension) will be excluded. Thoracostomy tube insertion for pleural drainage as sole thoracic procedure is excluded as this falls outwith the context of major pulmonary resection. Thoracostomy is included as a search term due to variable description in the literature and in select cases where associated with additional surgical entry to the thoracic cavity and/or OLV, to enable identification of relevant physiological studies.

3. **Comment:** *The intervention definition seems too broad to me. You include systemic vasoactive drugs alongside targeted pulmonary vasodilators. I suggest including these*

agents only when they were used to modify pulmonary haemodynamics or RV function.

Response: Thank you for this comment. We agree that several included agents, particularly B1-adrenoceptor agonists and inodilators, have systemic effects in addition to their pulmonary vasodilating properties. However, we have intentionally adopted a broad intervention definition because our primary purpose is to map the extent and nature of the available evidence rather than evaluate the efficacy of a narrowly defined intervention. From pilot searches, it has proven challenging to clearly identify an indication/rationale for agents with pulmonary vasodilating properties, particularly in case reports. Given our interest in identifying pharmacodynamic endpoints, a broader spectrum of agents which induce pulmonary vasodilation, even if used systemically, has been included. We are concerned that restricting eligibility only to studies that explicitly state pulmonary haemodynamic or RV-directed intent risks excluding relevant evidence and introducing subjective judgement during study selection.

We are aware, for example, of the existence of a significant literature using Nitric Oxide (NO) to improve oxygenation during OLV. Neglecting this literature would potentially exclude a cohort of papers which would be valuable to comment on the pharmacodynamic effects and safety of NO even if the intended pharmacological effect was not haemodynamic.

The reviewer makes an important point, however. To address this concern, we will extract and report interventions by pharmacological class and intended therapeutic use. This approach will allow readers to distinguish targeted pulmonary vasodilators

from agents with broader systemic effects whilst maintaining a comprehensive mapping of the evidence base, consistent with the review objectives.

The manuscript now reads:

- Page 17, lines 286-290:
    - Basic descriptive (e.g. lead author(s), publication year, country, study participants, surgery, pulmonary vasodilator(s) used, pharmacological class, indication for treatment (e.g. hypoxaemia, elevated pulmonary artery pressure), intended therapeutic use (e.g. prophylactic, therapeutic, rescue), route of administration, outcome measure, study type) data will be tabulated.

4. **Comment:** *Please define "perioperative administration". What time window will you accept, and will chronic pulmonary hypertension treatment simply continued during admission be eligible? I'd also distinguish prophylactic, therapeutic, and rescue use.*

Response: Thank you for this important question. We now specify a time window and address chronic PH treatment (see amended manuscript Page 12, Figure 1: Inclusion & Exclusion Criteria: Concept). Perioperative administration will be defined as "administration of any pulmonary vasodilating agent within 48 hours of surgery/procedure". Chronic PH medication which remains unchanged will not be included. However, escalation of a pulmonary vasodilating agent dose or introduction of a new pulmonary vasodilator in a PH patient already established on maintained pulmonary vasodilator therapy will be included. We will report on prophylactic, therapeutic and rescue use, where available.

The manuscript now reads:

- Inclusion criteria:
    - Perioperative administration of any pulmonary vasodilators/inodilators via any route
        - Perioperative defined as administration within 48 hours of surgery/procedure
        - Uptitrated dose or introduction of new pulmonary vasodilator in the perioperative period in patients on established chronic pulmonary hypertension treatment
- Exclusion criteria:
    - Studies involving administration of pulmonary vasodilators/inodilators in patients not undergoing surgery
    - Inhaled oxygen as the sole pulmonary vasodilator agent
    - Unchanged perioperative continuation of pulmonary vasodilators for chronic pulmonary hypertension

In addition, we distinguish between uses (see above changes applied in response to Comment 4: Page 17, lines 286-290).

5. **Comment:** *I'm a bit concerned that the outcome block in the search strategy may exclude relevant studies. Have you validated the strategy against known eligible studies? I'd consider removing this block.* **Response:** This block was strongly recommended by our information scientist colleagues to ensure the search incorporated adverse physiological effects of the intervention. The search was piloted using Embase and Medline including and excluding this block. The inclusion of this block was found to consistently include several key eligible papers we deemed essential for our review. The search terms here are intentionally broad.

6. **Comment:** *Riociguat is listed as eligible but does not appear in the search. I guess "betaprost" is a typo for beraprost. The appendices should present the final searches rather than pilot versions.*

Response: Thank you for noticing this typo, and the omission of Riociguat. The final search strategies have been amended and are confirmed in the appendices, which "betaprost" corrected to "beraprost". A search including the soluble guanylate cyclase stimulators "riociguat" and "vericiguat" on both Embase and Medline added no additional results. In addition, following further discussion we have concluded that Riociguat and Vericiguat are not relevant in the acute context (used only in specific subtypes of chronic pulmonary hypertension). Therefore, it has been removed from the manuscript (previously bulleted line 221).

7. **Comment:** *Please state how you'll handle non-English full texts, conference abstracts, protocols, and theses.*

Response: Our institution does not provide manual, in-house foreign language journal article translation. Non-English abstracts will therefore be excluded. Any records with available English abstracts entering full text screening without full texts available in English will be excluded on the same grounds (line 329). Grey literature (inc. conference abstracts, protocols and theses) is excluded for feasibility reasons, high duplication risk and anticipated lack of extractable physiological data (line 331-334).

8. **Comment:** *Please confirm that two reviewers will assess all full texts independently. I'd avoid requiring unanimous agreement, as this may unintentionally favour exclusion. Instead, use third-reviewer adjudication.*

Response: Thank you for highlighting this important omission. We have amended the manuscript accordingly to reflect our intended plan (reproduced below). Two authors will assess full texts independently. Unanimous agreement may unintentionally favour exclusion, therefore third-author adjudication will be used.

The manuscript now reads:

- Page 16, lines 273-284:
  - **Selection of sources of evidence.** Referenced results will be merged using EndNote 21 (Clarivate) software. Covidence (Veritas Health Innovation, Melbourne, Australia) software will be used to de-duplicate and screen results. Two independent authors (GL & AB) will screen for inclusion by title and abstract with disagreement arbitration by one of two independent subject-specialist co-authors (BS & PM). All abstracts selected for inclusion will be full text screened by inclusion/exclusion criteria by two independent authors. Authors will meet to consider disagreements or uncertainties in this secondary screening process. In the event of disagreement or uncertainty in this secondary screening process, a third subject-specialist reviewer will adjudicate. The systematic literature search will be represented using a PRISMA-ScR flowchart.

9. **Comment:** *I think the data-charting form is too limited for your objectives. I'd add baseline pulmonary hypertension and RV status, operation and resection extent, one-lung ventilation details, treatment indication, dose, timing, duration, comparator, outcome timing, adverse events.*

Response: Thank you for these suggestions. These characteristics have been added to the pilot data extraction tool, which now reads:

**Source**	**Population**		**Concept**	**Context***	**Results**
Citation & descriptors	Demographics<br>Baseline pulmonary hypertension<br>Right ventricular status (function, dimensions etc.)	Surgical characteristics<br>Operation/procedure<br>Resection extent (if lung resection)<br>One-lung ventilation characteristics	Pulmonary vasodilator(s) used & administration characteristics<br>Dose, timing & duration	Indication for pulmonary vasodilator<br>Treatment intent (prophylaxis, therapy, rescue)<br>Outcomes<br>Outcome timing<br>Effects<br>Comparator<br>Adverse events	Main findings

*Table 1 – **Pilot data extraction table.***

In describing our approach to data charting and data items, please also see Page 17, lines 290-297 which remain unchanged:

- This will be piloted following initial identification of key papers to test feasibility for final review and refined as necessary prior to final data extraction upon identification of literature for review. The pilot data extraction tool [Appendix 1: Table 1] may be amended following planned piloting on a purposive sample - 20% of included studies - in line with JBI guidance, an iterative process to enhance capture of variables required before commencing full extraction. The

final completed tool – and amendments, if present – will be included in the scoping review manuscript.

VERSION 2 - REVIEW

Reviewer 1

Name Lampridis, Savvas

Affiliation Imperial College London National Heart and Lung Institute, Faculty of Medicine

Date 28-Aug-2026

COI

Thank you for the revision. I think your manuscript has improved a lot and most of my previous comments have been addressed. I've just got a few remaining points:

1. I'm still not fully convinced by the very broad intervention definition. Including drugs like dobutamine, dopamine or adrenaline simply because they may have secondary pulmonary vasodilating effects risks bringing in studies that aren't really about pulmonary vasodilator therapy. I'd suggest defining a clearer eligibility rule for these agents.

2. I'm also still concerned about keeping the outcome block as a mandatory part of the search. Showing that several known eligible studies are retrieved doesn't really tell us whether relevant studies are missing. I'd either remove this block or validate the final strategy against a representative set of known eligible papers.

3. The surgical population is clearer now, but the eligibility criteria still aren't completely consistent. Please use the same definition, especially for one-lung ventilation, entry into the thoracic cavity and thoracostomy.

4. I'm not sure the rationale for excluding riociguat and vericiguat is fully consistent with the revised criterion allowing perioperative initiation or escalation of pulmonary hypertension therapy. Please make this consistent.

VERSION 2 - AUTHOR RESPONSE

__**Reviewer's comments to author**__

General response: Thank you once more for taking the time to review this revised manuscript. We are pleased that the reviewer found the revised manuscript to be improved and are grateful for the opportunity to further improve it.

In response to the reviewer's comments/queries:

1. **Comment:** *I'm still not fully convinced by the very broad intervention definition. Including drugs like dobutamine, dopamine or adrenaline simply because they may have secondary pulmonary vasodilating effects risks bringing in studies that aren't really about pulmonary vasodilator therapy. I'd suggest defining a clearer eligibility rule for these agents.*

Response: Thank you for the opportunity to further address this important point. We are concerned that excluding drugs with secondary vasodilating effects may miss an important opportunity to assess data relevant to the right ventricular-pulmonary vascular unit within the perioperative one-lung ventilation context. For example, the following record would not be obtained if secondary pulmonary vasodilators were excluded:

- Nomoto Y, Kawamura M. Pulmonary gas exchange effects by nitroglycerin, dopamine and dobutamine during one-lung ventilation in man. Can J Anaesth. 1989 May;36(3 Pt 1):273-7. doi: 10.1007/BF03010764. PMID: 2497996.

This paper however remains directly relevant to the review objectives because it examines adults undergoing one-lung ventilation and reports pulmonary vascular haemodynamic outcomes including pulmonary vascular resistance index and pulmonary artery pressure. Furthermore, it would be particularly important not to miss any relevant safety signal regarding use of any of the included drugs in the thoracic surgical population.

In order to improve methodological clarity, we have amended the existing eligibility criteria such that studies involving these drugs that do not assess relevant physiological outcomes (measures of: right ventricular function, right ventricular or pulmonary vascular pressure, right ventricular afterload, right ventricular-pulmonary arterial coupling) will be excluded.

To demonstrate the benefit of applying the new criteria, the following record would (appropriately) no longer be included for analysis following screening:

- Xia, R., Yin, H., Xia, Zy. *et al.* Effect of Intravenous Infusion of Dexmedetomidine Combined with Inhalation of Isoflurane on Arterial Oxygenation and Intrapulmonary Shunt During Single-Lung Ventilation. *Cell Biochem Biophys* 67, 1547–1550 (2013). https://doi.org/10.1007/s12013-013-9659-8.

This paper describes the use of Dexmedetomidine (which has secondary pulmonary vasodilating properties) in adults undergoing lung resection with one-lung ventilation, but no pulmonary vascular or right ventricular physiological outcomes are reported.

Furthermore, the following record would also not be included for analysis upon application of the newly proposed eligibility criteria, as it comments only on shunt fraction (oxygenation outcomes), rather than the haemodynamic outcomes of interest to our review.

- Wei, W., & Wang, M. (2011). Does small dose adrenaline administration benefit of oxygenation during one-lung ventilation?: 5AP2-4. European Journal of Anaesthesiology, 28, 73.

Thank you for permitting us the opportunity to add this important refinement to our scoping review protocol. The manuscript now reads (forthwith; amended/new text in red, unchanged text in **black**):

- Concept: pages 12-13, lines 212-221:
  - Pulmonary vasodilators are defined as drugs which primarily act on the pulmonary vasculature with a vasodilatory effect and include pulmonary inodilators. Given the relative novelty of this therapeutic approach (modulating pulmonary vascular physiology) in the context of thoracic surgery drugs which have a secondary pulmonary vasodilatory effect (e.g. Dobutamine) – which may be considered to have an alternative primary effect (e.g. positive inotropy) – are included provided haemodynamic outcomes measures of the right ventricular-pulmonary vascular unit are reported. Where records report the use of secondary pulmonary vasodilating drugs in adults undergoing one-lung ventilation but no right ventricular or pulmonary vascular haemodynamic measures are reported (e.g. modifications to oxygenation only), they will be excluded during screening. All routes of administration will be included including (but not limited to): inhaled, oral or intravenous (peripheral or central) route. Classes/drugs included (but not limited to) are:
    - Phosphodiesterase-3 & -5 inhibitors (e.g. Milrinone, Enoximone, Sildenafil, Tadalafil)
    - Inhaled nitric oxide
    - Prostacyclin analogues (Epoprostenol, Iloprost, Treprostinil)
    - Endothelin receptor antagonists (e.g. Bosentan)
    - Calcium sensitisers (e.g. Levosimendan)

        - Beta-1 adrenoceptor agonists (e.g. Dobutamine, Dopexamine, Adrenaline, Dopamine)

- Fig. 1. Scoping review inclusion and exclusion criteria
    - Exclusion criteria: Concept:
        - Studies involving administration of pulmonary vasodilators/ inodilators in patients not undergoing surgery
        - Inhaled oxygen as the sole pulmonary vasodilator agent
        - Unchanged perioperative continuation of pulmonary vasodilators for chronic pulmonary hypertension
        - Studies involving secondary pulmonary vasodilating agents where no right ventricular or pulmonary vascular haemodynamic measures are reported

This restriction is intentionally applied to secondary pulmonary vasodilators only, to avoid capturing studies in which these agents were administered primarily for unrelated indications and where pulmonary vascular effects were not evaluated.

2. **Comment:** *I'm also still concerned about keeping the outcome block as a mandatory part of the search. Showing that several known eligible studies are retrieved doesn't really tell us whether relevant studies are missing. I'd either remove this block or validate the final strategy against a representative set of known eligible papers.*

Response: We appreciate the opportunity to provide justification for this outcome block, which was considered in detail during the search strategy development process. Specialist information scientists (ER, AW & KC) assisted with search strategy development, all supporting this outcome block given the objectives set out in our scoping review protocol. As a pathway to a clinical trial of inhaled pulmonary

vasodilators in thoracic surgery, we are particularly interested in safety outcomes, including both RV-PA haemodynamic and other systemic effects, as these are likely to be highly relevant when developing a trial protocol and applying for funding and ethics. Our search strategy within this block is intentionally very broad.

Our piloting of the search strategy resulted in >5000 initial records (OVID Embase alone). Removing this block results in >9800 records, a nearly 100% increase. This would significantly impact feasibility of the scoping review. Comparing against recently published scoping reviews (inc. in BMJ Open) in interventional perioperative healthcare research, our search strategy already results in the higher range of the number of records entering first-stage screening.

Our search strategy was developed in line with a representative set of eligible papers. For example, the following papers deemed essential for inclusion were present when the "outcome" block of the search was present (OVID Embase):

- Kim, N., Lee, S. H., Joe, Y., Kim, T., Shin, H., & Oh, Y. J. (2020). Effects of Inhaled Iloprost on Lung Mechanics and Myocardial Function During One-Lung Ventilation in Chronic Obstructive Pulmonary Disease Patients Combined With Poor Lung Oxygenation. Anesthesia & Analgesia, 130(5), 1407–1414. https://doi.org/10.1213/ANE.0000000000004733
- Rocca GD, Passariello M, Coccia C, Costa MG, Di Marco P, Venuta F, Rendina EA, Pietropaoli P. Inhaled nitric oxide administration during one-lung ventilation in patients undergoing thoracic surgery. J Cardiothorac Vasc Anesth. 2001 Apr;15(2):218-23. doi: 10.1053/jcan.2001.21972. PMID: 11312483. Wilson WC, Kapelanski DP, Benumof JL, Newhart 2nd JW, Johnson FW, Channick RN.

    - Inhaled nitric oxide (40 ppm) during one-lung ventilation, in the lateral decubitus position, does not decrease pulmonary vascular resistance or improve oxygenation in normal patients. J Cardiothorac Vasc Anesth. 1997 Apr;11(2): 172-6. doi: 10.1016/s1053-0770(97)90209-3. PMID: 9105988.

We agree that retrieval of a small number of exemplar studies alone cannot formally establish search sensitivity. However, as this review concerns a relatively niche and poorly indexed field lacking established terminology, formal validation against a large gold-standard set is not feasible. Instead, we have undertaken iterative development with specialist information scientists and confirmed retrieval of studies representing differing drug classes, publication periods, and physiological outcome domains. In addition, reference-list screening of all included studies will be conducted to identify potentially missed records. We therefore believe the current strategy represents an appropriate balance between sensitivity and feasibility for a scoping review.

3. **Comment:** *The surgical population is clearer now, but the eligibility criteria still aren't completely consistent. Please use the same definition, especially for one-lung ventilation, entry into the thoracic cavity and thoracostomy.*

Response: Thank you for highlighting this inconsistency. The inclusion and exclusion criteria table has been amended accordingly. Further changes have been applied below the Rationale and Population sub-headings to ensure consistency.

The manuscript now reads:

- Fig. 1. Scoping review inclusion and exclusion criteria
    - Inclusion criteria: Population:
        - Adults (≥18 years)
        - Human only

        - Elective (planned) or emergency (unplanned) surgery
        - Lung resection and/or surgeries entering the thoracic cavity involving one-lung ventilation
        - Thoracotomy or thoracoscopic surgery
        - Upper gastrointestinal surgeries will be included where the thoracic cavity is entered
- Rationale (lines 117-120)
    - The intended surgical population of this review is those associated with acutely elevated RV afterload, namely lung resection and other surgeries entering the thoracic cavity that involve one-lung ventilation [replacing "namely one-lung ventilation and and/or those entering the thoracic cavity"].
        - Note: historical duplicated "and" and broader framing than revised eligibility criteria both corrected.
- Population (lines 193-199)
    - Adult (≥18 years old) humans undergoing planned (elective) and unplanned (emergency) thoracic surgery using either thoracotomy ["thoracostomy" removed] or thoracoscopy. For the purposes of the review, thoracic surgery will be defined as: lung resection and ["/or" removed] other surgeries entering the thoracic cavity that involve ["involving" removed] one-lung ventilation (also termed *lung isolation* and *selective pulmonary ventilation*). Studies involving mixed populations will be eligible if extractable data meets the inclusion criteria.

4. **Comment:** *I'm not sure the rationale for excluding riociguat and vericiguat is fully consistent with the revised criterion allowing perioperative initiation or escalation of pulmonary hypertension therapy. Please make this consistent.*

Response: Upon reflection, we agree with the reviewer's comment. Whilst records reporting use of these agents in the acute context is unlikely, they may be feasibly used in the semi-acute perioperative context in select patients. We now include both "riociguat" and "vericiguat" in the final search strategies, which are attached as supplementary files (see OVID Embase search line number 23 and OVID Medline search line number 24).
