# Supplementary material for: Use, physiological effects and safety of pulmonary vasodilator therapy in thoracic surgical patients: a scoping review protocol
Source: BMJ Open. 2026 Oct 8;16(10):e123437. doi: 10.1136/bmjopen-2026-123437 (PMC13656151; doi:10.1136/bmjopen-2026-123437)
Supplement: Supplementary file 2 [file bmjopen-16-10-s001.docx]

Supplementary Appendix 1. Pilot data extraction table.

| **Source** | **Population** | | **Concept** | **Context*** | **Results** |
| --- | --- | --- | --- | --- | --- |
| Citation & descriptors | Demographics  Baseline pulmonary hypertension  Right ventricular status (function, dimensions etc.) | Surgical characteristics  Operation/procedure  Resection extent (if lung resection)  One-lung ventilation characteristics | Pulmonary vasodilator(s) used & administration characteristics  Dose, timing & duration | Indication for pulmonary vasodilator  Treatment intent (prophylaxis, therapy, rescue)  Outcomes  Outcome timing  Effects  Comparator  Adverse events | Main findings |

Table 1 – **Pilot data extraction table.**

*Context fields are indicative and may be modified iteratively.
