# Supplementary material for: Use, physiological effects and safety of pulmonary vasodilator therapy in thoracic surgical patients: a scoping review protocol
Source: BMJ Open. 2026 Oct 8;16(10):e123437. doi: 10.1136/bmjopen-2026-123437 (PMC13656151; doi:10.1136/bmjopen-2026-123437)
Supplement: Supplementary file 3 [file bmjopen-16-10-s002.docx]

Supplementary Appendix 2. Final search strategy for Medline (via OVID) database.

# Ovid MEDLINE(R) ALL <1946 to August 23, 2026>

## Line number Search term

1	exp Vasodilator Agents/

2	((pulmonary adj1 vasodilat*) or inodilat*).ti,ab,kw,kf.

3	exp Nitric Oxide/

4	(nitric* adj1 oxid*).ti,ab,kw,kf.

5	3 or 4

6	exp Administration, Inhalation/ or exp Inhalation/ or inhal*.ti,ab,kw,kf.

7	5 and 6

8	exp Adrenergic beta-Agonists/ or (beta adj3 agon*).ti,ab,kw,kf.

9	exp Iloprost/ or exp Epoprostenol/ or (iloprost or epoprosten* or treprostin* or betaprost).ti,ab,kw,kf.

10	exp phosphodiesterase 3 inhibitors/ or exp phosphodiesterase 5 inhibitors/

11	exp Milrinone/ or exp Enoximone/

12	(milrinon* or enoximon*).ti,ab,kw,kf.

13	exp Sildenafil Citrate/ or exp Tadalafil/

14	(sildenaf* or tadalaf*).ti,ab,kw,kf.

15	exp Simendan/

16	(levosimend* or simend*).ti,ab,kw,kf.

17	(calcium adj1 sensit*).ti,ab,kw,kf.

18	exp Bosentan/ or exp Benzenesulfonamides/ or exp Endothelin Receptor Antagonists/

19	bosent*.ti,ab,kw,kf.

20	exp Dopamine/ or exp Dobutamine/

21	(dopamin* or dobutamin* or dopexamin*).ti,ab,kw,kf.

22	exp Epinephrine/

23	(epinephrin* or adrenalin*).ti,ab,kw,kf.

24	(riociguat or vericiguat).ti,ab,kw,kf.

25	1 or 2 or 7 or 8 or 9 or 10 or 11 or 12 or 13 or 14 or 15 or 16 or 17 or 18 or 19 or 20 or 21 or 22 or 23 or 24

26	exp One-Lung Ventilation/

27	(one adj1 lung adj1 an?esthes*).ti,ab,kw,kf.

28	"lung isolation".ti,ab,kw,kf.

29	(double adj1 lumen adj2 tub*).ti,ab,kw,kf.

30	((endobronch* or bronch*) adj1 block*).ti,ab,kw,kf.

31	((one or singl* or select*) adj1 (lung or pulmon*)).ti,ab,kw,kf.

32	(ventilat* or an?esthe*).ti,ab,kw,kf.

33	31 and 32

34	26 or 27 or 28 or 29 or 30 or 33

35	exp Thoracic Surgery/

36	(lung adj1 resect*).ti,ab,kw,kf.

37	((thora* or lung*) adj3 surg*).ti,ab,kw,kf.

38	(minimal* adj1 invas* adj1 (thorac* or lung) adj1 surg*).ti,ab,kw,kf.

39	exp Pneumonectomy/ or exp Thoracic Surgery, Video-Assisted/ or exp Thoracotomy/ or exp Thoracostomy/ or exp Thoracoscopy/

40	(pulmonect* or pneumonect* or thoracot* or thoracos*).ti,ab,kw,kf.

41	(wedge adj1 resect*).ti,ab,kw,kf.

42	((lung or pulmon*) adj2 segmentect*).ti,ab,kw,kf.

43	segmentect*.ti,ab,kw,kf.

44	(lung* or pulmon* or video* or VATS* or robot* or open*).ti,ab,kw,kf.

45	lobect*.ti,ab,kw,kf.

46	44 and 45

47	exp Esophagectomy/

48	(esophagect* or oesophagect*).ti,ab,kw,kf.

49	((esophag* or oesophag*) adj2 resect*).ti,ab,kw,kf.

50	35 or 36 or 37 or 38 or 39 or 40 or 41 or 42 or 43 or 46 or 47 or 48 or 49

51	34 or 50

52	ae.fs.

53	tolera*.mp.

54	((pulmon* or physiologic* or vascul* or h?emodynam* or system*) adj5 (effect* or outcome* or impact* or improv*)).mp.

55	exp Treatment Outcome/ or exp "Drug-Related Side Effects and Adverse Reactions"/ or exp Drug Tolerance/ or exp pharmacokinetics/ or exp dose-response relationship, drug/ or exp drug interactions/

56	((drug* or treat* or therap*) adj5 (outcome* or effect* or efficacy or impact*)).mp.

57	52 or 53 or 54 or 55 or 56

58	25 and 51 and 57

59	limit 58 to english language
