# Supplementary material for: Use, physiological effects and safety of pulmonary vasodilator therapy in thoracic surgical patients: a scoping review protocol
Source: BMJ Open. 2026 Oct 8;16(10):e123437. doi: 10.1136/bmjopen-2026-123437 (PMC13656151; doi:10.1136/bmjopen-2026-123437)
Supplement: Supplementary file 4 [file bmjopen-16-10-s003.docx]

Supplementary Appendix 3. Final search strategy for Embase (via OVID) database.

# Embase 1947-Present, updated daily

## Line Number Search term

1	exp vasodilator agent/

2	((pulmonary adj1 vasodilat*) or inodilat*).ti,ab,kw,kf.

3	exp nitric oxide/

4	(nitric* adj1 oxid*).ti,ab,kw,kf.

5	3 or 4

6	exp inhalation/ or exp inhalational drug administration/ or inhal*.ti,ab,kw,kf.

7	5 and 6

8	exp beta adrenergic receptor stimulating agent/ or (beta adj3 agon*).ti,ab,kw,kf.

9	(iloprost or epoprosten* or treprostin* or betaprost).ti,ab,kw,kf.

10	exp phosphodiesterase III inhibitor/ or exp phosphodiesterase V inhibitor/

11	exp milrinone/ or exp enoximone/

12	(milrinon* or enoximon*).ti,ab,kw,kf.

13	exp sildenafil nitrate/ or exp sildenafil/ or exp tadalafil/

14	(sildenaf* or tadalaf*).ti,ab,kw,kf.

15	exp levosimendan/ or exp simendan/ or exp calcium sensitizer/

16	(levosimend* or simend*).ti,ab,kw,kf.

17	exp bosentan/ or exp benzenesulfonamide derivative/ or exp endothelin receptor antagonist/

18	bosent*.ti,ab,kw,kf.

19	exp dopamine/ or exp dobutamine/ or exp dopexamine/

20	(dopamin* or dobutamin* or dopexamin*).ti,ab,kw,kf.

21	exp epinephrine/

22	(epinephrin* or adrenalin*).ti,ab,kw,kf.

23	(riociguat or vericiguat).ti,ab,kw,kf.

24	1 or 2 or 7 or 8 or 9 or 10 or 11 or 12 or 13 or 14 or 15 or 16 or 17 or 18 or 19 or 20 or 21 or 22 or 23

25	"lung isolation".ti,ab,kw,kf.

26	(one adj1 lung adj1 an?esthes*).ti,ab,kw,kf.

27	exp one lung ventilation/ or exp double lumen tube/ or exp endobronchial blocker/

28	(double adj1 lumen adj2 tub*).ti,ab,kw,kf.

29	((endobronch* or bronch*) adj1 block*).ti,ab,kw,kf.

30	((one or singl* or select*) adj1 (lung or pulmon*)).ti,ab,kw,kf.

31	(ventilat* or an?esthe*).ti,ab,kw,kf.

32	30 and 31

33	25 or 26 or 27 or 28 or 29 or 32

34	thorax surgery/ or lung surgery/ or exp minimally invasive thorax surgery/ or exp thoracoscopic surgery/

35	(lung adj1 resect*).ti,ab,kw,kf.

36	((thora* or lung*) adj3 surg*).ti,ab,kw,kf.

37	(minimal* adj1 invas* adj1 (thorac* or lung) adj1 surg*).ti,ab,kw,kf.

38	exp pneumonectomy/ or exp wedge resection/ or exp segmentectomy/ or exp video assisted thoracoscopic lobectomy/ or exp lung lobectomy/ or exp robot assisted thoracoscopic lobectomy/ or exp thoracotomy/ or exp thoracostomy/ or exp thoracoscopy/

39	(pulmonect* or pneumonect* or thoracot* or thoracos*).ti,ab,kw,kf.

40	(lung* or pulmon* or video* or VATS* or robot* or open*).ti,ab,kw,kf.

41	lobect*.ti,ab,kw,kf.

42	exp lobectomy/

43	41 or 42

44	40 and 43

45	exp minimally invasive esophagectomy/ or exp McKeown esophagectomy/ or exp thoracoscopic esophagectomy/ or exp transthoracic esophagectomy/ or exp transhiatal esophagectomy/ or exp esophagectomy/ or exp laparoscopic esophagectomy/

46	(esophagect* or oesophagect*).ti,ab,kw,kf.

47	((esophag* or oesophag*) adj2 resect*).ti,ab,kw,kf.

48	34 or 35 or 36 or 37 or 38 or 39 or 44 or 45 or 46 or 47

49	33 or 48

50	exp treatment outcome/ or exp drug effect/ or exp therapy effect/ or exp adverse event/ or side effect/ or exp drug efficacy/ or exp drug tolerance/ or exp pharmacodynamics/ or exp pharmacokinetics/ or exp dose response/ or exp drug interaction/

51	ae.fs.

52	tolera*.mp.

53	((pulmon* or physiologic* or h?emodynamic* or vascul* or system*) adj5 (effect* or outcome* or impact* or improv*)).mp

54	((drug* or treat* or therap*) adj5 (outcome* or effect* or efficacy or impact*)).mp.

55	50 or 51 or 52 or 53 or 54

56	24 and 49 and 55

57	limit 56 to english language
